# Characterization of TPP-binding proteins in Methanococci archaeal species

**DOI:** 10.6026/97320630012359

**Published:** 2016-11-22

**Authors:** Laura K. Harris

**Affiliations:** 1 Department of Science, Davenport University, Lansing, Michigan, United States of America; 2 Department of Health Informatics, Rutgers School of Health Professions, Newark, New Jersey, United States of America

**Keywords:** Characterization, TPP- binding proteins, Methanococci species

## Abstract

Acetolactate synthase (ALS) is a highly conserved protein family responsible for producing branched chain amino acids. In Methanocaldococcus jannaschii, two ALS proteins, MJ0277 and MJ0663 exist though variations in features between them are noted. Researchers are quick to examine MJ0277 homologs due to their increased function and close relationship, but few have characterized MJ0663 homologs. This study identified homologs for both MJ0277 and MJ0663 in all 15 Methanococci species with fully sequenced genomes. EggNOG database does not define four of the MJ0663 homologs, JH146_1236, WP_004591614, WP_018154400, and EHP89635. BLASTP comparisons suggest these four proteins had around 30% identity to MJ0277 homologs, close to the identity similarities between other MJ0663 homologs to the MJ0277 homologous group. ExPASY physiochemical characterization shows a statistically significant difference in molecular weight and grand average hydropathy between homologous groups. CDD-BLAST showed distinct domains between homologous groups. MJ0277 homologs had TPP_AHAS and PRL06276 while MJ0663 homologs had TPP_enzymes super family and IlvB domains instead. Multiple sequence alignment using PROMALS3D showed the MJ0277 homologs a tighter group than MJ0663 and its homologs. PHYLIP showed these homologous groups as evolutionarily distinct yet equal distance from bacterial ALS proteins of established structure. The four proteins EggNOG did not define had the same features as other MJ0663 homologs. This indicates that JH146_1236, WP_004591614, WP_018154400, and EHP89635, should be included in EggNOG database cluster arCOG02000 with the other MJ0663 homologs.

## Background

The Methanococci class of archaeal organisms currently consists of 15 coccoid methanogens according to the National Center for Biotechnology Information (NCBI). Methanococcus jannaschii was the first fully sequenced archaea, leading to interesting revelations on the similarities across domains [1]. M. jannaschii, a hyperthermophilic organism isolated from the base of a hydrothermal vent on the East Pacific Rise that emits lighter-hued minerals containing barium, silicon, and calcium, grows best at 85oC with high pressure [2]. The National Center of Biotechnology Information (NCBI) re-classified it to the Methanocaldococcus genus alongside six other Methanococcus organisms due to their ability to thrive at high temperatures alongside a low 16S rRNA sequence similarity with their five-mesophilic relatives that retained the Methanococcus name. According to the UCSC Genome Browser, M. jannaschii and M. fervens are close, followed by M. vulcanius, M. infernus, and then M. igneus [3]. M. aerolicus forms a separate branch that includes M. vannielii and M. maripaludis. M. okinawensis fits in between M. jannaschii and M. aerolicus while M. voltae groups with M. aeolicus. Interestingly, M. thermolithotrophicus has similar 16S rRNA sequence similarity to Methanococcus organisms, except it is thermophilic. Together they make up the Methanococcaceae family. According to NCBI, the thermophilic nature of M. thermolithotrophicus connects the mesophilic Methanococcus genus to their two thermophilic Methanotorris relatives.

When M. jannaschii was sequenced, open reading frame numbers 0277 and 0663 corresponding to locations relative to the ori, were assigned as genes encoding large sub-units of acetohydroxy acid synthase (EC 4.1.3.18, AHS) based on the algorithm by NCBI called the Basic Local Alignment Search Tool (BLAST). AHS assists with the production of branched chain amino acids: leucine, isoleucine, and valine [4,5]. Currently for M. jannaschii DSM2661, NCBI currently states that MJ0277 and MJ0663 are acetolactate synthase (ALS) large subunits. The Gene Ontology Consortium shows AHS and ALS (EC 2.2.1.6) to be synonymous [6]. ALS belongs to a superfamily of thiamine pyrophosphate (TPP)-dependent enzymes capable of catalyzing a variety of reactions. No one has determined the structure of archaeal ALS, but x-ray crystal structures are available for Klebsiella pneumoniae (1OZF) and Bacillus subtilis (4RJI) in the Protein Data Bank.

ALS is highly conserved across domains. Bowen showed through phylogenetic analysis that AHS (ALS) diverged from the other TPPbinding enzymes prior to the split between archaeal and bacterial lineages [7]. Therefore, it is not surprising that researchers detect AHS (ALS) activity in the cell extracts from several Methanococci species including Methanococcus aeolicus, Methanococcus maripaludis, and Methanococcus voltae [8,9,10]. However, data from several researchers suggest that MJ0277 and MJ0663 are different from each other. Phylogenetic studies show MJ0277 and an AHS (ALS) from Methanococcus aeolicus related to AHS (ALS) proteins from bacterial and eukaryotic species more closely than to MJ0663 and MJ0663 did not look related to other bacterial or eukaryotic TPP-binding proteins like AHS (ALS) or pyruvate oxidase [7]. Garder showed that the amino acid sequence for MJ0277 was more similar than MJ0663 when compared to ilvB in Methanococcus maripaludis, 72.9% and 31.4%, respectively [10]. Because of these differences, Universal Protein Resource (UniProt) currently calls MJ0663 an uncharacterized protein whereas MJ0277 reads as ilvB.

The MJ0277 protein and its homologs have received much attention due to their clear membership in the ALS protein family. Because of its differences, MJ0663 has not received the same focus so to date there are no studies on the homologs of MJ0663 in the literature. However, the EggNOG 4.5 database of orthologous groups and functional annotation shows MJ0663 as belonging to two clusters of archaeal orthologous groups (COG): COG0028 and arCOG02000 (TPP-binding proteins). COG0028 has over 5000 ALS proteins from more than 1700 species across domains whereas arCOG02000 had proteins from 11 Methanococci species. NCBI taxonomy currently lists 15 Methanococci species. If MJ0663 and its homologs are part of a conserved protein family, as EggNOG suggests, there should be identifiable homologs in the four species not currently included in the EggNOG database.

The purpose of this study is to use in silico methods to identify and characterize homologs of either MJ0277 or MJ0663 in Methanococci species. Since there are notable differences between MJ0277 and MJ0663 and prior research suggests they belong to two different, yet related, protein families, any new homologs should have similar observable differences. These analyses would confirm current information about these protein sub-families, further the understanding of the relatedness of ALS-related TPP-binding proteins in Methanococci archaeal species, and improve public database accuracy.

## Methodology

Both protein sequences for MJ0277 and MJ0663 underwent a NCBI protein-protein BLAST (BLASTP) with each individual Methanococci species to identify homologous groups. Table 1 lists the identified homologs from each organism. The sequences for all Table 1 proteins plus ALS proteins from Klebsiella pneumoniae and Bacillus subtilis, Protein Data Bank entries 1OZF and 4RJI, respectively were downloaded from NCBI.

The Expasy Protparam server calculated several physicochemical characterizations for each protein including number of amino acids, amino acid composition and frequencies, molecular weight, and the total number of charged residues (aspartic acid plus glutamic acid for positively charged and the sum of arginine and lysine for negatively charged) [11]. From that, the program calculates the theoretical isoelectric point, which is the pH where a molecule carries no net electrical charge. The algorithm also determines the amount of light a protein absorbs at a 280nm wavelength also known as the extinction coefficient, which is helpful for purification procedures [12]. ExPASy calculated the relative volume of a protein occupied by open side chain amino acids as the aliphatic index [13]. The grand average hydropathy (GRAVY) is the sum of hydropathy values of all amino acids in the protein divided by the number of resides [14]. Therefore, GRAVY relates to the extent of hydrophobicity for a given molecule. Minitab calculated the statistical significance using the Chi-squared (c2) Goodness of Fit (one variable) analyses.

Both Pfam and the conserved domain database (CDD) identified domains. Pfam is a comprehensive collection of multiple sequence alignments and Hidden Markov Models (HMMs) that represent protein domains and families [15,16]. PfamA is a set of manually curated and annotated models each based on a seed alignment and an automatically created full alignment. The seed alignment contains a group of proteins in the same family while the full alignment contains all noticeable protein sequences belonging to the family as defined by HMMs searches of primary sequence databases. Within NCBI lies another complimentary program for domain identification. The CDD is searchable using a protein query via the CD-Search interface. This algorithm uses Reversed Position Specific BLAST (RPS-BLAST), a Position-Specific Iterative (PSI)-BLAST variant, to establish position-specific scoring matrices with the protein sequence [17]. Together, Pfam and CDD-BLAST examine protein domains.

PROMALS3D used whole protein sequences in FASTA format for three multiple sequence alignments, one with MJ0277 homologs only, one with MJ0663 homologs only, and one with all proteins in Table 1 plus the Klebsiella pneumoniae (1OZF) and Bacillus subtilis (4RJI). The analyses used PROMALS3D’s default settings [18].

Similarly, ClustalW aligned all Table 1 proteins plus 1OZF and 4RJI whole protein sequences in FASTA format for input into the PHYLIP package Protdist program to produce a distance matrix using default settings such as the Jones-Taylor-Thornton matrix distance model [19,20]. Neighbor, another program in the PHYLIP suite, used this matrix to construct a neighbor joining and unweighted pair group method with arithmetic mean trees. The Fitch-Margoliash and Least-Squares Distance method, another phylogenetic tree building approach, verified the results. The program DrawTree illustrated all phylogenetic trees.

## Discussion

Of the 15 Methanococci species with genomes available on NCBI, there was an identifiable homolog for both MJ0277 and MJ0663 in every species, listed in Table 1. The MJ0663 homologs for four species, Methanocaldococcus bathoardescens, Methanocaldococcus villosus, Methanothermococcus thermolithotrophicus, and Methanotorris formicicus, were new proteins not included in EggNOG arCOG02000. When MJ0277 was compared with its homologs, they achieved an average 99% query coverage (SD +1%) with 81% identity (SD +11%) to the target protein sequence. When MJ0277 and its homologs were compared to MJ0663, they averaged 97% query coverage (SD +1%) with 29% identity (SD +0.4%). Alternatively, when MJ0663 was compared with its homologs, they achieved an average 97% query coverage (SD +3%) with 64% identity (SD +13%) to the target protein sequence. When MJ0663 and its homologs were compared to MJ0277, they averaged 92% query coverage (SD +3%) with 31% identity (SD +4%). These results demonstrate that MJ0277 and its homologs have a stronger sequence similarity than MJ0663 and its homologs do and that the two groups look different at a protein sequence level.

### Physiochemical Characterization

Table 2 summarizes several physiochemical characterizations. MJ0277 and homologs averaged 595 amino acids (SD +8) while MJ0663 and homologs averaged 506 amino acids (SD +26, p=0.298). Molecular weight reflected similar findings (64951 +941 versus 56498 +2653 for the MJ0277 and MJ0663 groups, respectively, p=0.000). The groups had similar theoretical isoelectric point averages (p=1.000). This was not surprising because the MJ0277 and MJ0663 groups had an average amino acid composition of 12.4% and 12.1% for negatively charged amino acids alongside 10.8% and 11.4% for positively charged residues. Extinction coefficients and aliphatic index between the groups were unremarkable (p=1.000), but there was a difference in hydrophobicity as seen in the average GRAVY results (-0.05 +0.03 versus -0.23 +0.08 for the MJ0277 and MJ0663 groups, respectively, p=0.000). These results indicate that the two protein families are similar in physiochemical properties but have some identifiable differences in molecular weight and hydrophobicity.

### Domain Characterization

Both Pfam and CDD-BLAST algorithms identified domains for these TPP-binding proteins. Pfam-A results did not show a difference between the groups. All proteins except METIN_RS00550 had Thiamine pyrophosphate enzyme N-terminal binding, central, and C-terminal binding domains corresponding to clans CL0254, CL0085, and CL0254, respectively. Protein METIN_RS00550 was missing the TPP enzyme N-terminal domain, but had the other two domains.

The CDD-BLAST database identified a difference between the groups. While CDD-BLAST assigned TPP_PYR_POX_like and TPP_enzyme_M domains to all proteins regardless of group, MJ0277 and its homologs had TPP_AHAS and PRK06276 domains whereas MJ0663 and its homologs had TPP_enzymes super family and IlvB domains instead. The TPP_enzyme_M domain came from the Pfam database, so it is interesting that Pfam itself did not assign this domain to METIN_RS00550, yet CDD-BLAST did. Both of the two domains specific to MJ0277 and its homologs were ALS related. The TPP_AHAS domain referred to the AHS (ALS) subfamily of TPP-binding proteins, comprised of proteins similar to the large catalytic subunit of AHAS [21]. NCBI defines PRK06276 as an ALS catalytic subunit. The domains specific to MJ0663 and its homologs were not as function specific. The TPP_enzymes super family domain simply referred to these proteins having TPP-binding module found in many key metabolic enzymes that use TPP as a cofactor. The IlvB domain indicated an ALS large subunit or other TPP-requiring enzyme related to amino acid or coenzyme transport and metabolism.

### Sequence Alignment

**Figure 1:**
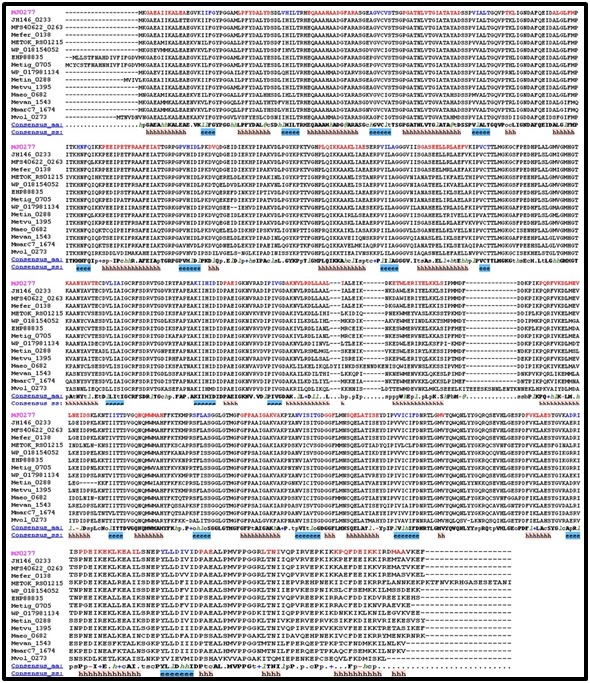
Alignment of MJ0277 homologs aligned by PROMALS3D. Magenta names are representative sequences colored red to identify predicted alpha-helix secondary structures. The black names belonging to the same alignment group as the magenta name above it, indicating a strong relationship between the two. Consensus_aa, consensus amino acid sequence; Consensus_ss, consensus predicted secondary structures; h, consensus predicted secondary structure alpha-helix.

**Figure 2:**
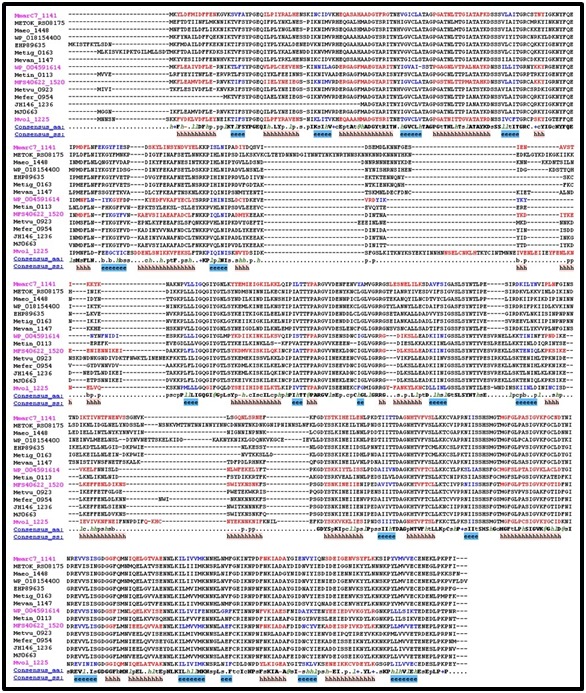
Alignment of MJ0663homologs aligned by PROMALS3D. Magenta names are representative sequences colored red to identify predicted alpha-helix secondary structures. The black names belonging to the same alignment group as the magenta name above it, indicating a strong relationship between the two. Consensus_aa, consensus amino acid sequence; Consensus_ss, consensus predicted secondary structures; h, consensus predicted secondary structure alpha-helix.

MJ0277 and its homologs were aligned together and separate from MJ0663 and its homologs. Figure 1 and Figure 2 shows the multiple alignment data for separate homologous group alignments. MJ0277 and its homologs are more conserved than MJ0663 is with its homologs as seen by the magenta color illustrating representative alignment sequences. This is further illustrated comparing consensus sequences between groups. In both groups, the N-terminus is more closely conserved than the rest of the protein as seen by the consensus sequence. The consensus sequence for the MJ0663 homologous group becomes ill defined for the central and C-terminus whereas the consensus sequence for MJ0277 homologous group is well defined throughout the protein.

### Phylogeny Characterization

**Figure 3:**
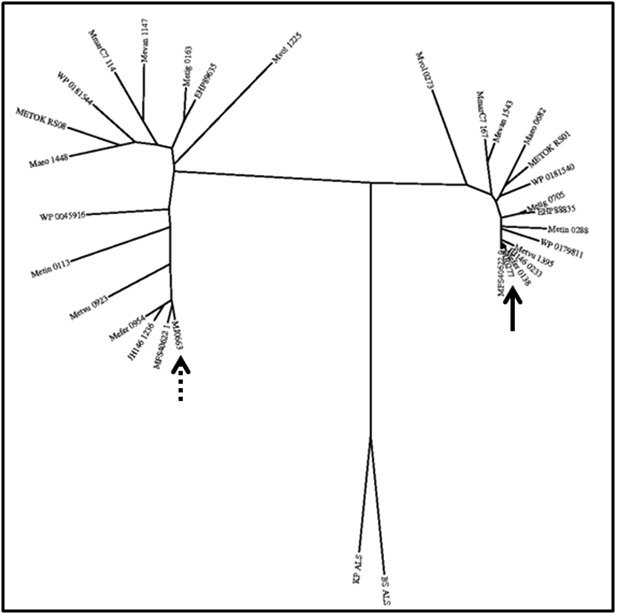
Representative phylogenetic tree of proteins produced via PHYLIP package programs showing members of the acetolactate synthase (ALS) family. Nomenclature of genes is consistent with Table 1. The solid arrow highlights MJ0277 while the dashed arrow points to MJ0663. This illustrates that MJ0277 and MJ0663 are closely related to their respective homologs from other Methanococci species, but are different from each other. Both groups are equally distant from experimentally established bacterial ALS proteins.

To examine phylogenetic relatedness, various PHYLIP programs analyzed a CLUSTALW alignment of all 30 proteins and two bacterial ALS proteins with established structure to produce phylogenetic trees. Different algorithms with Protdist were used, as were different tree building methods. All trees looked similar to Figure 3, illustrating how related MJ0277 and its homologs are to each other yet are distant to MJ0663 at its homologs with both groups equal distance to bacterial ALS proteins. These results support those from PROMALS3D.

## Conclusion

For each of 15 Methanococci species with genomes available on NCBI, there was an identifiable homolog for both MJ0277 and MJ0663. Four MJ0663 homologs JH146_1236, WP_004591614, WP_018154400, and EHP89635 from species Methanocaldococcus bathoardescens, Methanocaldococcus villosus, Methanothermococcus thermolithotrophicus, and Methanotorris formicicus, respectively, are proteins not included in EggNOG database cluster arCOG02000. BLASTP comparisons suggest these homologs had a 30% identity to MJ0277 homologs, similar to identity similarities between other MJ0663 homologs to the MJ0277 homologous group. ExPASy characterization showed the physiochemical chemical properties such as molecular weight and GRAVY are significantly similar among MJ0663 homologs but not MJ0277 homologs. CDD-BLAST identified two domains common among all MJ0663 homologs that are not present in MJ0277 homologs and vice versa. MJ0277 homologs had TPP_AHAS and PRL06276 while MJ0663 homologs had TPP_enzymes super family and IlvB domains instead. Multiple sequence alignment analysis showed all MJ0277 homologs as closely related but there are subtle differences among MJ0663 homologs. The consensus sequence for the MJ0663 homologous group becomes ill defined for the central and C-terminus whereas the consensus sequence for MJ0277 homologous group is well defined throughout the protein. PHYLIP illustrated MJ0277 and its homologs as phylogenetically related but the group is separate from their conserved MJ0663 relatives. Both homologous groups are equally distant to bacterial ALS proteins of established structure. These results support those from multiple sequence alignment. Ergo, the four MJ0663 homologs identified here, JH146_1236, WP_004591614, WP_018154400, and EHP89635, should be included in EggNOG database cluster arCOG02000.

## Figures and Tables

**Table 1:** A dataset of Methanococci archaeal proteins studied

Name of organism including strain	NCBI Protein Locus Tag		NCBI Assigned Function
	New	Old	
Methanocaldococcus jannaschii DSM 2661	Not applicable	MJ0277	ALS large subunit
	Not applicable	MJ0663	ALS large subunit
Methanocaldococcus bathoardescens JH146	JH146_RS01170	JH146_0233	ALS large subunit
	JH146_RS06320	JH146_1236	ALS large subunit
Methanocaldococcus sp. FS406-22	MFS40622_RS01340	MFS40622_0263	ALS large subunit, biosynthetic
	MFS40622_RS07655	MFS40622_1520	TPP-binding domain protein
Methanocaldococcus fervens AG86	MEFER_RS00680	Mefer_0138	ALS large subunit, biosynthetic
	MEFER_RS04855	Mefer_0954	TPP-binding domain protein
Methanocaldococcus vulcanius M7	METVU_RS06835	Metvu_1395	ALS large subunit, biosynthetic
	METVU_RS04510	Metvu_0923	TPP-binding domain protein
Methanocaldococcus infernus ME	METIN_RS01430	Metin_0288	ALS large subunit, biosynthetic
	METIN_RS00550	Metin_0113	TPP-binding domain protein
Methanococcus aeolicus Nankai-3	MAEO_RS03395	Maeo_0682	ALS large subunit, biosynthetic
	MAEO_RS07420	Maeo_1448	TPP-binding domain protein
Methanococcus	MVOL_RS01405	Mvol_0273	ALS large subunit, biosynthetic
voltae A3	MVOL_RS06100	Mvol_1225	TPP-binding domain protein
Methanococcus	MMARC7_RS08600	MmarC7_1674	ALS large subunit, biosynthetic
maripaludis C7	MMARC7_RS05925	MmarC7_1141	TPP-binding domain protein
Methanococcus	MEVAN_RS07925	Mevan_1543	ALS large subunit, biosynthetic
vannielii SB	MEVAN_RS05905	Mevan_1147	TPP-binding domain protein
Methanocaldococcus villosus KIN24-T80	WP_017981134	Not applicable	ALS
	WP_004591614	Not applicable	TPP-binding protein
Methanothermococcus thermolithotrophicus DSM 2095	WP_018154052	Not applicable	ALS
	WP_018154400	Not applicable	Hypothetical protein
Methanotorris formicicus Mc-S-70	EHP88835	Not applicable	ALS large subunit, biosynthetic
	EHP89635	Not applicable	TPP-binding domain protein
Methanothermococcus okinawensis IH1	METOK_RS01215	Metok_0247	ALS large subunit, biosynthetic
	METOK_RS08175	Metok_1612	ALS
Methanotorris igneus Kol 5	METIG_RS03470	Metig_0705	ALS large subunit, biosynthetic
	METIG_RS00805	Metig_0163	ALS
			
ALS, acetolactate synthase; NCBI, National Center for Biotechnology Information; TPP, thiamine pyrophosphate.			

**Table 2:** Physiochemical properties of acetolactate-related proteins

Protein	# AA	MW	pI	# neg	# pos	EC	II	AI	GRAVY
MJ0277	591	64492.8	5.49	78	65	40720	36.93	98.58	-0.002
JH146_RS01170	591	64560.9	5.5	78	65	40590	37.98	97.75	-0.021
MFS40622_RS01340	591	64496	5.68	77	66	39230	35.84	98.26	-0.004
MEFER_RS00680	591	64218.5	5.49	78	65	40590	36.5	97.26	-0.002
METVU_RS06835	591	64549.7	5.76	76	66	39100	39.22	97.39	-0.042
METIN_RS01430	590	64476.8	6.03	75	68	42080	37.07	99.19	-0.034
MAEO_RS03395	599	65394.6	6.23	68	62	38110	37.64	98.05	-0.067
MVOL_RS01405	601	65259.9	5.55	74	57	29260	36.42	96.57	-0.082
MMARC7_RS08600	587	64000.9	6.09	64	57	42080	31.23	92.44	-0.072
MEVAN_RS07925	587	64123.1	6.61	63	60	39100	37.04	94.24	-0.079
METOK_RS01215	608	66440.8	6.06	72	64	42330	40.86	95.02	-0.061
METIG_RS03470	608	66601	5.78	76	65	43950	37.36	95.02	-0.044
METVI_RS0106325	587	64652	5.89	78	69	42080	37.72	101.19	-0.068
WP_018154052	590	64198.5	5.93	73	64	39230	36.58	94.08	-0.052
EHP88835	608	66805.1	5.71	78	66	42330	36.57	95.79	-0.074
MJ0663	494	55354.9	6.04	62	60	37290	30.45	97.27	-0.146
JH146_RS06320	491	49223.5	6.29	53	52	31330	32.07	98.84	-0.2
MFS40622_RS07655	490	55076.5	5.77	64	60	32820	38.06	94.71	-0.189
MEFER_RS04855	477	53199.2	6.12	59	57	35800	32.95	94.8	-0.202
METVU_RS04510	511	57855.4	5.79	71	65	39230	34.29	92.86	-0.276
METIN_RS00550	477	54119.1	5.62	69	62	33160	37.19	97.86	-0.228
MAEO_RS07420	507	56638.2	5.26	56	46	39240	34.8	97.69	-0.329
MVOL_RS06100	552	62645.8	6.03	68	63	43750	30.9	94.09	-0.273
MMARC7_RS05925	501	55762.6	5.64	56	48	37630	23.6	92.79	-0.16
MEVAN_RS05905	501	56082	6.43	52	49	32040	26.98	88.92	-0.165
METOK_RS08175	568	64269.2	8.62	57	64	49670	36.3	92.32	-0.415
METIG_RS00805	512	57183.3	7.03	62	62	34310	25.94	100.33	-0.126
METVI_RS0106795	478	54569.7	6.02	61	58	52190	37.57	100.13	-0.201
WP_018154400	527	59466.1	5.88	65	59	43710	34.59	97.13	-0.266
EHP89635	502	56024.4	5.7	63	58	38780	26.39	95.72	-0.211
# AA, number of amino acids; MW, molecular weight; pI, theoretical isoelectric point; # neg, total number of negatively charged residues (Asp + Glu); # pos, total number of positively charged residues (Arg + Lys); EC, extinction coefficient assuming all pairs of Cys residues form cystines; AI, aliphatic index; GRAVY, grand average hydropathy.
